# Ultrasonic Quality Assurance at Magnesia Shotcrete Sealing Structures

**DOI:** 10.3390/s22228717

**Published:** 2022-11-11

**Authors:** Vera Lay, Ute Effner, Ernst Niederleithinger, Jennifer Arendt, Martin Hofmann, Wolfram Kudla

**Affiliations:** 1 Bundesanstalt für Materialforschung und Prüfung, 12205 Berlin, Germany; vera.lay@bam.de (V.L.); ute.effner@bam.de (U.E.); 2 Technical University Bergakademie Freiberg, 09596 Freiberg, Germany; jennifer.arendt@mabb.tu-freiberg.de (J.A.); martin.hofmann@mabb.tu-freiberg.de (M.H.); wolfram.kudla@mabb.tu-freiberg.de (W.K.)

**Keywords:** ultrasonics, imaging, engineered barrier, underground, shotcrete, magnesia concrete

## Abstract

Engineered barriers are a key element to enable safe nuclear waste disposal. One method currently under research for their construction is magnesia concrete applied in a shotcrete procedure. In this study, the ultrasonic echo method is evaluated as a means for quality assurance. Imaging of internal structures (backwall, boreholes) and defects, such as delamination, has successfully been achieved in the shotcrete. Additionally, detailed information about the potential cause of selected reflectors are obtained by phase analysis. In several test blocks of various sizes, no consistent concrete section boundaries have been found by ultrasonic imaging, which was verified by subsequent drilling and complementary tests. An experiment with artificial defects imitating cracks, air-filled voids, and material with lower density has been challenging and shows the limitations of the current methods. Although significant defects, such as a large delamination, are reliably identified, several smaller defects are not identified. Generally, ultrasonic imaging provides a suitable base as a mean for quality assurance during and after the construction of sealing structures. However, further developments are required to enhance the reliability of the method and a full validation is still pending. Still, the method has potential to increase the safety of nuclear waste repositories.

## 1. Introduction

Nuclear waste disposal is an important task for society. In Germany, a decision towards the best possible location for a final repository for high level nuclear waste has been envisaged until 2031. All three potential geological types of host rock environments (salt rock, crystalline rock, clay) were analysed in detail concerning existing geological barriers. At the same time, complementary technical barriers safely protecting the waste were further developed. For salt as a host rock forming the given mountains, engineered barriers made of salt concrete were planned to safely seal underground galleries. They are also used in existing disposal sites [1,2].

Safe closure of underground disposal facilities and future high active waste repositories require appropriate building and sealing materials. Commonly, salt is a major threat to standard concrete if there is a risk of contact with magnesium-containing solutions. Thus, specifically tailored magnesia concrete with similar chemical characteristics to the surrounding salt host rock was used [3]. Moreover, constructions without reinforcement were planned to avoid corrosion. Decades of experience have led to stable salt and magnesia concretes [4,5,6,7], showing no significant chemical reactions with the salt rock while still fulfilling the needed compressive strength and low permeability. However, improving the production and workability of these materials is still a matter of further development. Ongoing research analyses the characteristics of magnesia concrete used at engineered barriers constructed as either mass concrete or shotcrete [8]. In this study, the dry-mix shotcrete procedure was used with magnesia concrete to ease the constructions procedures. The aim was to create reliable engineered barriers while providing sufficiently good underground applicability [3,7,9].

For standard mass concrete, ultrasonic measurements reliably serve as a tool to identify internal structures and major defects, such as delamination or cracks [10,11,12,13,14,15]. Here, we analyse the potential for applying ultrasonics as a quality assurance method at underground barrier systems made of shotcrete. The applicability of ultrasonic methods, with relatively deep penetration, has been proven [16]. For sealing structures made of shotcrete, it is of particular interest to ensure good coupling between concrete section boundaries to allow for the integrity of the sealing. As bad coupling between concrete section boundaries would resemble a delamination, the shotcrete and contained concrete section boundaries are analysed by the ultrasonic echo method, which is sensitive to occurring delaminations.

So far, to the best of our knowledge, only a few applications have been reported regarding shotcrete structures. The work reported often aims at analysing specific physical parameters, such as compressive strength [17], or is specifically applied at tunnel constructions, e.g., [18,19].

In this contribution, we show ultrasonic data acquired at shotcrete test bodies produced to analyse the applicability of magnesia shotcrete for engineered barriers in nuclear waste disposal. The aim is to analyse the potential for ultrasonic investigations to serve as a quality assurance tool to identify cracks or delamination. For this, we present the analysis of data measured using a unique deep penetration ultrasonic system at a large-scale test block to evaluate the capability of the ultrasonic method. Complementary small-scale experiments using a commercial ultrasonic echo device were performed. Furthermore, a blind test was executed with artificial defects (e.g., voids, notches) to test the capability of the ultrasonic investigations.

## 2. Materials and Methods

### 2.1. Experiments at Teutschenthal Mine with Magnesia Shotcrete

Teutschenthal mine is located close to Halle in Eastern Germany. The mine is run by GTS Grube Teutschenthal Sicherungs GMBH & Co. KG (Teutschenthal, Germany) and is a former potash salt mine (founded 1905), now working as a waste filling mine. In parts of the mine, scientific experiments with magnesia shotcrete are carried out under in situ underground conditions.

Within the project CARLA (“**CAR**nallititische **L**angzeitsichere **A**bdichtbauwerke”), three closure elements were installed in large-scale tests in the Carnallit Mountains in the Teutschenthal mine for demonstration of their technical feasibility and suitability [7]. The large-scale test 2 (named “Großversuch (GV2)”) was produced from magnesia concrete using the dry-mix shotcrete procedure. The shotcrete consisted of hard stone (quartz) aggregate, magnesia, and a salt solution (saturated with NaCl, containing MgCl_2_). The 10.25 m long structure consisted of 104 concreting sections with an average thickness of 9.9 cm. This magnesia shotcrete was a promising building material for drift-sealing elements in rock salt formations, especially with regard to the low permeability after spraying and the decreasing permeability after contact with solution.

**Figure 1:**
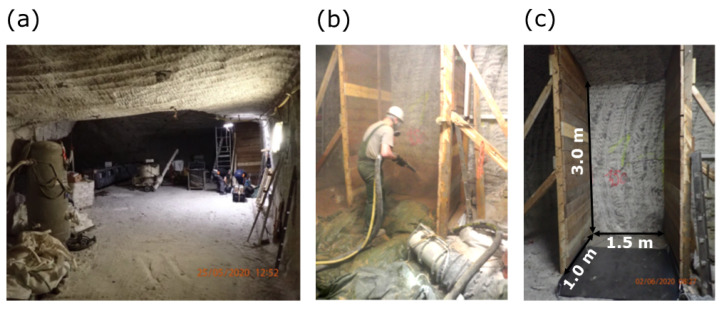
Impressions from shotcrete experiments at Teutschenthal mine: (**a**) experimental underground site, (**b**) during shotcrete procedure at a shotcrete box, (**c**) preparation of a shotcrete box.

In a new project “MgO-S³ - MgO-Spritzbeton für Streckenverschlusselemente für HAW-Endlager im Steinsalz”, i.e., ”Magnesia shotcrete for drift sealing elements for HAW repositories in salinar”, additional investigations were carried out on this magnesia shotcrete to further expand the knowledge level [9]. For this purpose, four tests were carried out in situ. At a height of 3 m and a width of 1.5 m, about 1 m of shotcrete was sprayed (Figure 1). A range of 4 to 6 layers with thicknesses between 12 to 30 cm were sprayed per test (named “Großspritzbetonversuch (GSBV)”). The first and second in situ shotcrete test aimed at reproducing the shotcrete formulation of GV2 from the previous project CARLA with different settings and other equipment (e.g., nozzle). For the following two tests, the hard stone aggregate was replaced by salt aggregate [9]. In all cases, the aggregate size was 0–8 mm. An overview of the experiments discussed here is summarised in Table 1. Drill cores were obtained from boreholes. Gas permeability was measured on the cores and in the boreholes in situ. Particularly, the potential effect of the concreting section boundaries is analysed in detail.

### 2.2. Ultrasonic Echo Method

**Figure 2:**
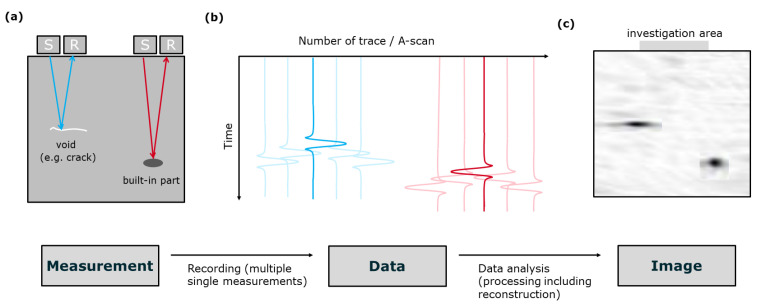
Summary of the applied ultrasonic echo method: (**a**) measurements with sender/receiver pair, (**b**) data represented in the time/space domain, (**c**) reconstructed ultrasonic image in the depth/space domain.

The ultrasonic echo method is widely used in non-destructive testing [10,11,13,14]. Ultrasonic transducers excite ultrasonic waves (sender S in Figure 2a) that travel through the inspected concrete. At defects such as cracks or embedded objects/voids, the ultrasonic waves are reflected and can be recorded again at receiving transducers at the surface (receiver R).

During the measurement, sender and receiver locations of the transducers are systematically moved along the surface in small steps to gather information from the entire investigation area. Time series of the received ultrasonic signals are recorded (Figure 2b), and obstacles are visible as reflections. Here, for simplicity, only the reflections of the indicated crack and built-in object are sketched. These data are then analysed in more detail to reconstruct the internal structures within the concrete (Figure 2c).

### 2.3. Ultrasonic Data Analysis

The acquired ultrasonic data is analysed by a synthetic aperture focussing technique (SAFT) implemented in the software InterSAFT [20,21]. From the recorded reflections in the time-domain, the imaging algorithms calculate the true locations of reflectors in the space-domain. This migration method is based on a diffraction stack technique, resulting in constructive interference of individual recorded ultrasonic signals at the location of reflectors within the structure. The actual imaging is done in 3D, and results are presented as slices from the reconstructed 3D volume.

The processing scheme includes zero time correction, geometry setting, and a specifically chosen band pass filter (here, 10–100 kHz) before performing the imaging procedure itself. For the reconstruction, a constant velocity was used that was previously determined by transmission measurements at cement cores produced for this purpose during the concreting of each specific shotcrete experiment. Mean velocities of v ${}_{s}$ (GV2) = 2430 m/s, v ${}_{s}$ (GSBV3) = 2270 m/s, and v ${}_{s}$ (GSBV4) = 2190 m/s were used for the respective test blocks.

Commonly, the reconstructed images of the Hilbert-transformed data are shown as absolute amplitudes to highlight the existing reflectors and allow easy interpretation. The focus of the presented analysis is to illustrate relative anomalies caused by reflective areas. Hence, the exact values of the absolute amplitudes were not comparable between different investigation areas as neither amplitude-preserving processing nor a relative correction of the amplitudes were applied here. Thus, the exact values of the plotted absolute amplitudes varied between individual data sets due to several occurring differences, such as attenuation, coupling, and the specific measurement geometry. With the applied processing, a comparison between individual data sets could only be done phenomenologically and not quantitatively.

However, additional information could be obtained with a specific phase analysis, taking into consideration the characteristics of the reflected wavefield [20,21]. This way, the impedance contrast could be determined. Negative impedance contrasts were associated with a reflection caused by “softer” material than the surrounding concrete, such as air, whereas positive impedance contrasts occurred at “denser” materials, such as steel. This evaluation provides an indication for material type/quality and/or possible defects.

### 2.4. Ultrasonic Data Acquisition

**Figure 3:**
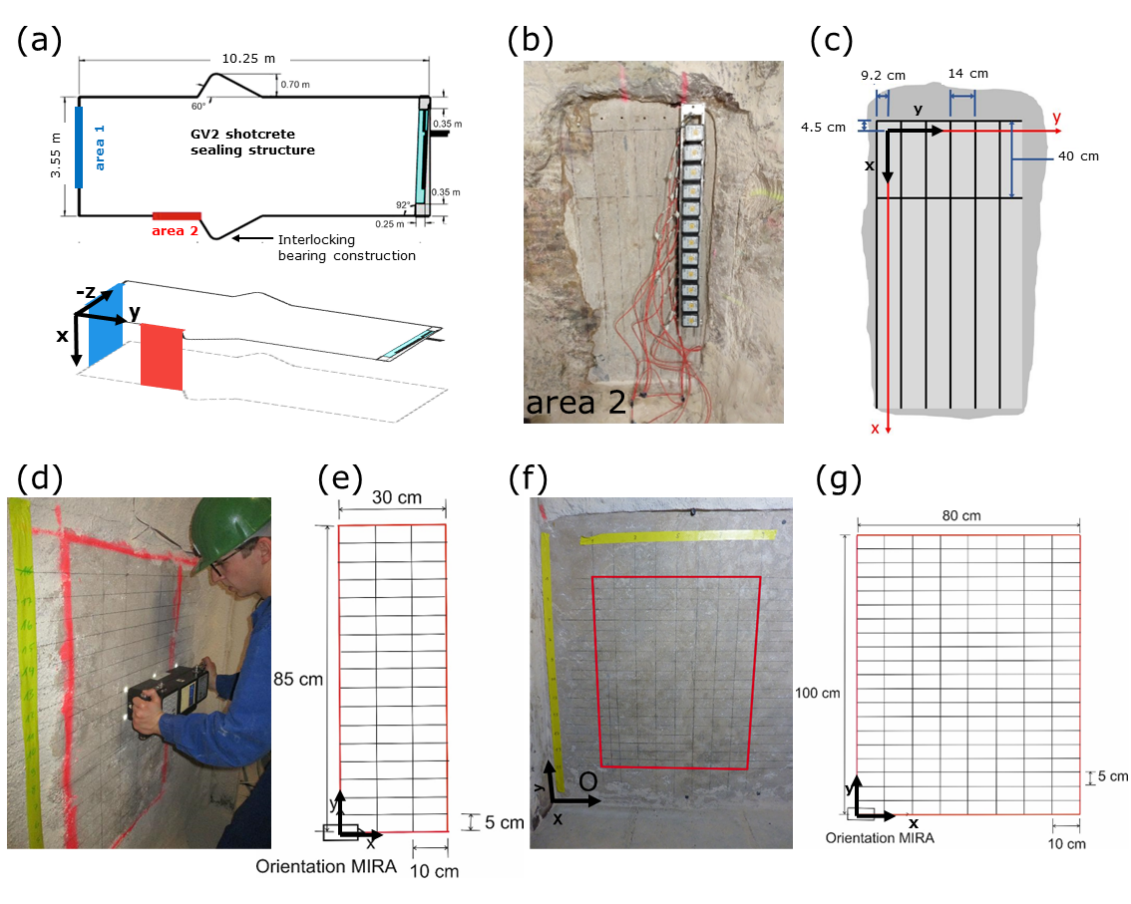
Ultrasonic data acquisition with (**a**–**c**) LAUS device and (**d**–**g**) array device. (**a**) Geometry GV2 experiment; (**b**) measurements at area 2 with LAUS; (**c**) corresponding measurement grid; (**d**) GSBV3 experiment with (**e**) corresponding measurement grid; (**f**) GSBV4 experiment with (**g**) corresponding measurement grid.

The ultrasonic data shown here were acquired with two different ultrasonic systems, as shown in Figure 3, and discussed in detail in [16,22]. Both devices used point contact shear wave transducers to avoid the need of a coupling agent and to reduce unwanted wave type conversion inside the structure, which would blur the images. For both systems, the measurement areas had to be smoothed by grinding before the measurements for better coupling. A rectangular grid was marked on the surface used to assist the measurements and to allow an accurate combination of the instrument locations.

First, the large aperture ultrasonic system “LAUS” was used [15]. In total, 12 units were operated at a frequency of 25 kHz, allowing a penetration depth of up to 9.6 m in salt/magnesia concrete [15,16,23]. Each of the 12 units consists of 32 combined ultrasonic transducers that worked as a transmitter or receiver. The spacing between individual units was 11 cm, resulting in a total length of ∼120 cm covered by a single LAUS measurement.

A first experiment was conducted from the frontal face of the large scale experimental block GV2 (area 1, blue in Figure 3a) in a previous project [7,16]. The measurement area was parallel to the shotcrete layers so that the ultrasonic wave travelled perpendicular to the shotcrete layers. Additional measurements from the sides were carried out here (area 2, red, in Figure 3a, specific details in Figure 3b,c). Hence, the wave propagation was parallel to the shotcrete layers. In total for area 2, the vertically oriented LAUS measured at 2 horizontal positions (spacing 40 cm) along 6 vertical lines (spacing 14 cm). Due to the measurement geometry, the setup was insensitive in the uppermost ∼20 cm and reflectors could not be detected here. With the dominant operating frequency of 25 kHz and an estimated ultrasonic shear wave velocity of v ${}_{s}$ (GV2) = 2430 m/s, the resulting dominant wavelength was about 9.7 cm. Usually, objects smaller than half of the wavelength, e.g., ∼5 cm here, cannot be resolved.

Second, a commercial system (A1040 MIRA manufactured by Acoustic Control Systems (ACS)) was used for the investigations at the smaller testblocks produced for this project (see Table 1). It consisted of a linear array of similar point contact transversal transducers, as in the LAUS system (but lower in number and more densely spaced). In total, 48 individual transducers measured on 12 linear arrays for the A1040 MIRA. Each linear array consisted of four individual transducers, and the lines had a distance of 3 cm, resulting in a whole array length of 33 cm. A working frequency of 50 kHz was applied after initial in situ tests, resulting in dominant wavelengths of 4.5 cm (GSBV3) and 4.4 cm (GSBV4). The measurements were taken at the front side of the construction, thus being parallel to the shotcrete layers with the ultrasonic wave travelling perpendicular to the layers. Measurements for both device orientations (i.e., also polarisation directions) were performed, but only results for horizontal device orientation are shown in the following.

Investigations at the shotcrete experimental block (GSBV3) were carried out along horizontal lines, taking individual measurements every 10 cm (see Figure 3d,e). In total, 18 horizontal lines with a spacing of 5 cm were occupied so that the measurements covered a central area as wide as 0.3 m × 0.85 m.

For the latest experimental block (GSBV4, Figure 3f,g), a total area of 0.8 m × 1.0 m was covered. In total, 21 horizontal lines with a line spacing of 5 cm were used for the horizontal device mode. At the upper half of the covered area, artificial defects were incorporated between the concrete layers. Their locations were unknown to the ultrasonic experts at first but revealed later for evaluation.

## 3. Results

### 3.1. Ultrasonic Imaging at Great Depths

**Figure 4:**
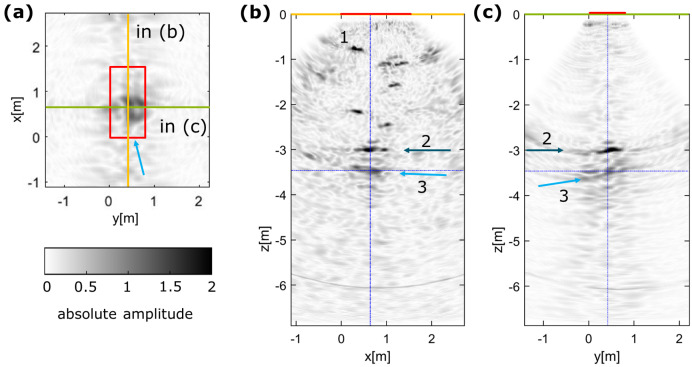
LAUS ultrasonic imaging results at area 2 at large scale shotcrete construction. (**a**) xy–slice at a depth of z = −3.5 m with measurement area (red) and corresponding slices marked. (**b**) yz–slice and (**c**) xz–slice.

For the large constructed barriers (GV2, ~10 m long), deeply penetrating ultrasonic investigations with the LAUS were conducted from the front [7,16] and from the side [9,22]. The measurements from area 2 marked in Figure 3a–c) are summarised in Figure 4.

The exemplary ultrasonic images in Figure 4 show the capacity of the LAUS when analysing the engineered barrier system from the side (area 2 in Figure 3a–c). Although the opposite side of the sealing structure is expected at z ≈ −3.5 m, data was recorded for the full length of the A–scans, also providing deeper information from within the salt rock environment. Although the self-healing nature of salt rock makes additional larger cracks unlikely, potential excavation-induced flaws can be monitored, particularly before the healing could take place. Distinct individual reflectors are clearly identified (number 1 in Figure 4b). The deepest reflector is identified at a depth of z = −3.5 m (light blue arrow, 3) with a slightly shallower reflector located at z = −3.0 m (dark blue arrow, 2). The reflectors 2 and 3 can be attributed to the bearing of the barrier system in the rock mass or internal flaws and the opposite wall of the structure.

**Figure 5:**
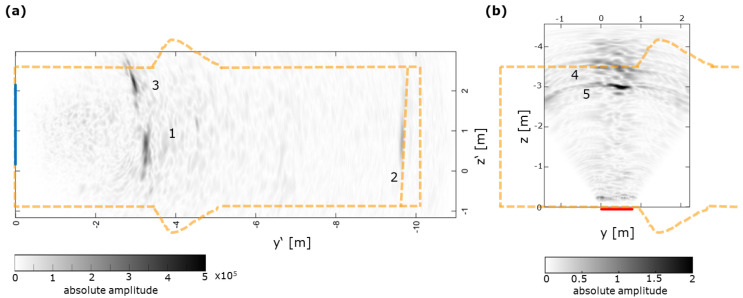
LAUS GV2 ultrasonic images (after [16]) and comparison to engineered barrier (orange dashed line, details in Figure 3a): (**a**) Ultrasonic imaging from the front (LAUS area 1, blue), results from [16] and (**b**) ultrasonic imaging from the side (LAUS area 2, red), equivalent to Figure 4c.

Figure 5 combines the previous results, measuring with the LAUS from the front ([16], Figure 5a), and the results presented here ([22], Figure 5b). The geometry of the sealing structure is indicated as orange dashed line according to the sketch provided in Figure 3. In Figure 5a, the reflector 1 is associated to the bearing at y’ = −3.3 m whereas reflector 3 might potentially be caused by a 3D effects of the bearing or an internal flaw. Clearly, reflector 2 can be associated to the location (y’ = −9.6 m) of a pressure chamber at the end of the sealing structure. Consistently, the backwall (i.e., the opposite side of the sealing structure) can be identified as a reflector when measuring from the side (number 4 in Figure 5b). Despite a slight local misalignment, the reflector number 5 might coincide with reflector 3 potentially caused by an internal flaw. Alternatively, reflector 5 might be associated to borehole drilled after the initial ultrasonic measurements in area 1 (blue).

### 3.2. Ultrasonic Imaging at Shotcrete Boxes

**Figure 6:**
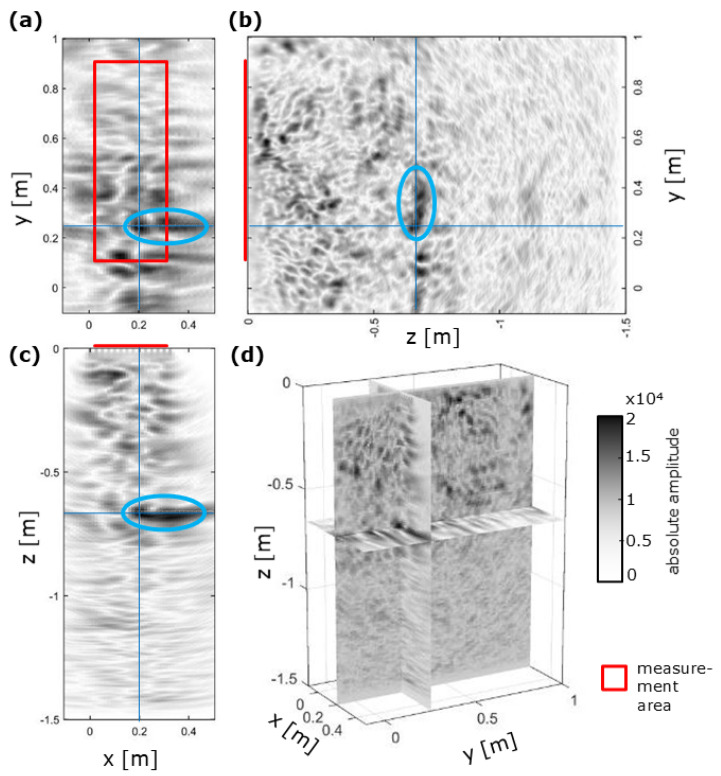
Ultrasonic imaging results of GSBV3 (after [22]) showing a distinct reflector (blue ellipse): (**a**) xy-slice (parallel to measurement surface), (**b**) corresponding yz-slice, (**c**) corresponding xz-slice, and (**d**) 3D overview of the presented slices.

For the shotcrete box experiment GSBV3, ultrasonic images showed several reflectors within the concrete body (Figure 6). Generally, the final images showed inhomogeneous structures that might have been associated with the characteristics of the shotcrete material and the higher frequency conventional ultrasonic measurement device. Nevertheless, prominent reflectors, such as the elongated reflector at z = −0.7 m in Figure 6, were clearly identified. Concreting sections were not systematically imaged so that a successful concreting was assumed as also indicated by observed low permeabilities measured on core samples in the laboratory. Moreover, no clear reflection of the backwall (at z ≈−1 m) was visible due to either higher general reflectivity of the shotcrete or very similar characteristics between the shotcrete and the salt rock, resulting in a low impedance contrast not causing a significant reflection.

### 3.3. Analysis of Potential Reflector Origin with Ultrasonic Phase Analysis

**Figure 7:**
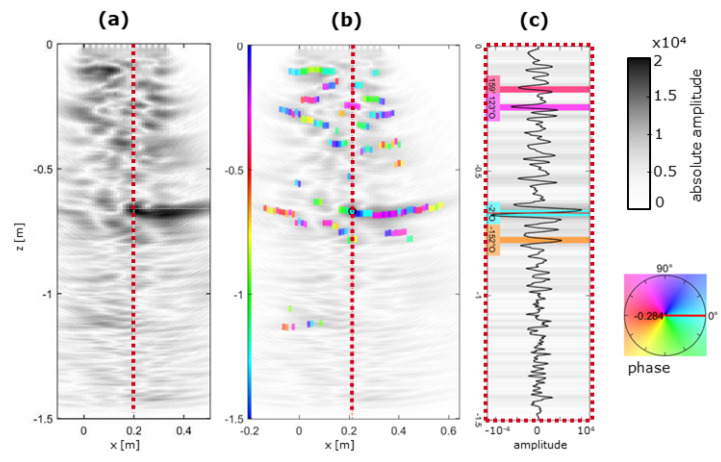
Phase analysis at GSBV3: (**a**) Ultrasonic image, as shown in Figure 6c, (**b**) overlain by phase analysis results; (**c**) individual analysed trace at central location (red dashed line) with determined phases of major reflectors.

As a detailed phase analysis of prominent reflectors may provide insight into the nature of the reflector, it was carried out for prominent reflectors here. Thus, their origin may be determined. At the experimental shotcrete box GSBV3, the results of the phase analysis are summarised in Figure 7. The analysed xz–trace (marked in Figure 7a) coincided with the significant reflector from Figure 6. Associated phases after analysis, according to [20], are plotted in Figure 7b, with details of the exact location (red dotted line) extracted in Figure 7c. The specific reflector at z = −0.7 m had a phase close to 0° and was associated with a negative impedance contrast.

Thus, the origin of the reflector was a weaker structure with an acoustic impedance lower than that of the surrounding magnesia shotcrete being caused by, e.g., cavities, cracks, or delamination. Later drilling showed a well-manufactured shotcrete, with some minor irregular cavities (opening 0.1–0.5 mm, length 1–5 cm) also at the respective depth that also caused the core to break at exactly that depth.

### 3.4. Blind Test with Artificial Defects

**Figure 8:**
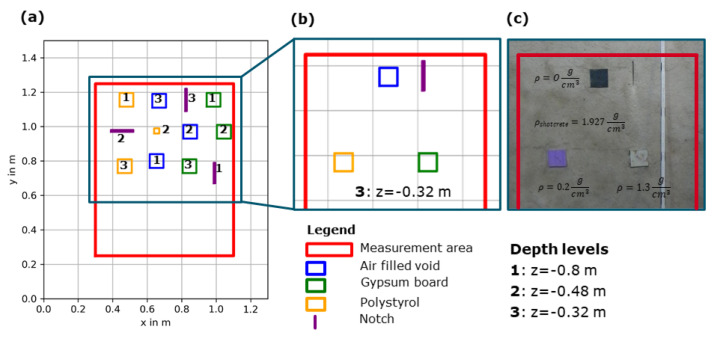
Experimental setup for GSBV4, including artifical defects: (**a**) Overview of all incorporated defects; (**b**) defects at the specific depth slice z = 0.32 m; (**c**) photo of the defects during construction at the same depth slice.

GSBV4 provides a unique experiment with several incorporated artificial defects [9]. Within a part of the area foreseen for ultrasonic measurements, four different type of defects were incorporated during the construction: air-filled void, polystyrol, gypsum board, and notches carved into the surface (Figure 8). The air-filled void was created by a 3 mm thin plastic casing. Most artefacts have a size of 8 cm and thickness of 3 cm and are located at three distinct depth levels, each being a concrete section boundary. The notches were carved as wide as 0.5–1 cm in the uppermost shotcrete layer but not filled so that partial re-filling by the following shotcrete layer cannot be excluded. The experimental setup of the shallowest depth layer (z = −0.32 m) is shown in Figure 8b,c.

**Figure 9:**
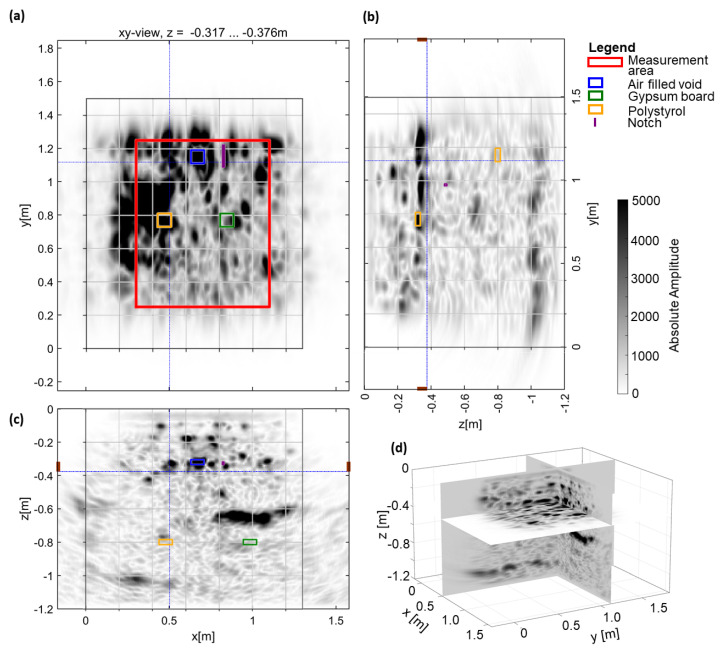
Ultrasonic imaging results of GSBV4 overlain by expected location of artificial defects (compare Figure 8): (**a**) xy–slice at z = −0.32 m, (**b**) yz–slice, (**c**) xz–slice, and (**d**) 3D view at marked slices.

In a first step, the ultrasonic data was analysed as a blind test without any prior information about the location, depth, or nature of the artefacts. A standard measurement layout with a linear array instrument was used with the standard procedures, concerning, for example, grid line distance. The results of the depth layer shown in detail for the experimental setup in Figure 8 are summarised in Figure 9a, with corresponding xz– and yz–slices.

The ultrasonic imaging result shows that many reflectors are imaged that hint to varying material characteristics. Without prior knowledge, structures not artificially incorporated are clearly identified, but not all artificial flaws introduced in the experiment are detected, as shown by the overlay with the actual locations of the artificial defects in Figure 9. During this analysis, a few indications were identified. The expected backwall (at z ≈−1 m) is partly visible in Figure 9, where the echo is not shadowed by shallower reflectors. Due to the generally inhomogeneous reflectivity of the shotcrete, some indications could later be correlated with the defects; but particularly, the deeper test flaws with lower contrast (e.g., gypsum board imitating a concrete with lower density) could not be seen, even when their location was known. Shallower artificial flaws and particularly the air-filled voids can be associated with reflectors. To verify the most significant reflectors, such as the strong reflector at z = −0.64 m between x–locations 0.75 m and 1.25 m, in Figure 9c, detailed phase analysis and subsequent drilling were undertaken.

### 3.5. Verification with Boreholes

**Figure 10:**
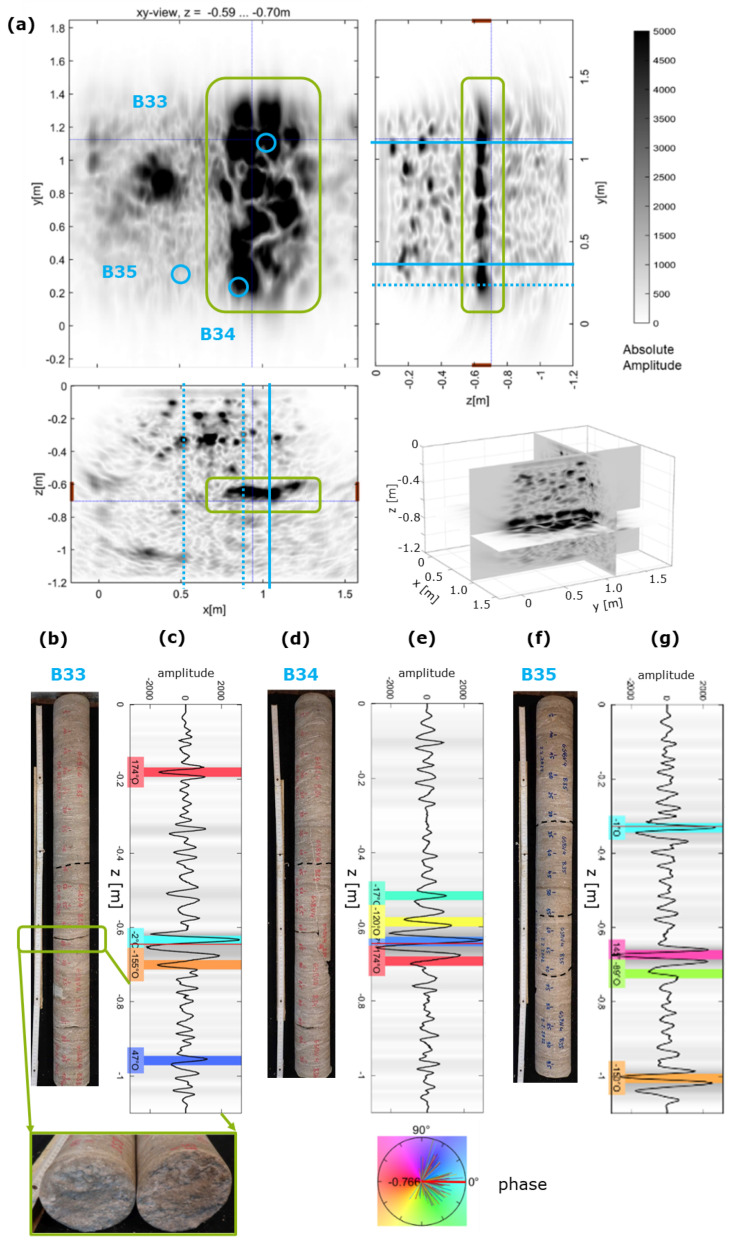
Ultrasonic imgaging results of GSBV4: (**a**) equivalent to Figure 9 at a summed depth interval of z = 0.59–0.7 m. Note the location of drilled boreholes (blue) and large reflector (green rectangle). (**b**,**d**,**f**) taken cores with (**c**,**e**,**g**) corresponding phase analysis at the same location.

During the previous experiments, no extensional reflectors were identified that were directly imaged at the known concreting boundaries. However, for GSBV4, the analysis of the ultrasonic imaging shows a major reflector that covers nearly half of the investigated area, as shown in Figure 10. Corresponding xz– and yz–slices also show the extent of this large reflector. Note that the location of verification boreholes [24] are marked (blue circles/lines) but are partly too far away from the plotted 2D–slices (dashed blue lines) to draw direct conclusions.

The depth slice, shown in Figure 10a, is summed over a range of z = −0.59…−0.7 m to enhance the reflector, being most prominent at depths between z = −0.62…−0.66 m. This depth correlates well with a concreting boundary between the second and third 16 cm thick concrete layers, i.e., at z = −0.64 m. Most of the energy is reflected at this depth so that no imaging of deeper structures is possible at this location. Due to the strong appearance of the reflector, ground truthing was done through phase analysis and drilled boreholes with cores taken (see core photos in Figure 10b,d,f). The borehole diameter was 107 mm. The taken core samples were located within the large ultrasonic anomaly (B33, B34) and one outside (B35) and helped to confirm and understand ultrasonic signals. Partly, failure caused by the drilling occurred and is marked by dashed lines. Most interestingly, the core B33 in Figure 10b shows a clear delamination (green rectangle) at a depth of z = −0.63 m (also see detailed photo of the fracture surface) and is also visible in a camera analysis of the borehole. Although the core B34 (Figure 10d) was also broken at z = −0.61 m, respectively, there was no widespread delamination detected but several smaller inhomogeneities.

At the locations of the boreholes, phase analysis after [20] was done equivalently to results discussed in Figure 7, with the ultrasonic measurements recorded before drilling. Here, Figure 10c,e,g each show the trace with determined phases at the location of the boreholes. The phases are colour-coded, with blue colours associated with negative impedance contrasts, i.e., sound weaker material, such as air, and red colours associated with positive impedance contrasts, i.e., sound stronger material, such as denser concrete.

For major reflectors in the phase analysis, there was a good correlation to the structures identified at the cores, although not all individual structures were assignable. A detailed analysis of the drilled boreholes by [24] shows a set of defects in the cores that can only partly be related to clear reflectors identified in the ultrasonic images. However, the determined phase associated with potentially air-filled voids coincides with the visible delamination (B33, Figure 10b,c). The coincidence of the analysed phases with drilled results was particularly strong for large reflectors with relatively high amplitudes and showed the value of the phase analysis.

## 4. Discussion

### 4.1. Applicability of Ultrasonics as a Quality Assurance Method at Shotcrete Engineered Barriers

In general, the experiments show the potential of ultrasonic methods to identify major structures within the manufactured magnesia shotcrete barriers. In general, shotcrete, compared to mass concrete, seemed to cause higher internal diffuse reflectivity, resulting in higher noise levels in ultrasonic data. Thus, reflectors of different internal structures can clearly be imaged, but not all artificial defects are detectable. Depending on the used main frequencies and the given sound velocities, the resulting wavelength is ∼4.4 cm at GSBV4, containing the artifical defects. This wavelength increases with depth due to attenuation effects. Hence, the detection of small (artifical) flaws is even more challenging.

Overall, major delaminations (size exceeding tens of centimeters) are reliably identified. Unexpected large-scale delaminations, a major potential risk for the integrity of these structures, are clearly imaged, even with commercially available equipment applied, with standard procedures analysing the upper meter. Thus, we conclude that ultrasonic quality assurance forms a valid basis to be implemented in quality assessment programs.

However, there are currently still limits. The measurement areas have to be grinded for good coupling, which generates significant cost and delay, consequently affecting the manufacturing process accordingly. Even for deep penetration measurement devices, such as the LAUS, it would be necessary to have regular tests after several concreting steps. Structures made of salt or magnesia concrete have so far never been imaged by ultrasound deeper than 9.6 m depth [16], but actual barriers might be significantly larger.

### 4.2. Influence of Ultrasonic Velocities

Generally, a constant ultrasonic shear wave velocity is used for the reconstruction of ultrasonic echo data assuming that the concrete’s elastic features are largely homogeneous. This approach usually provides robust results and is also used here with velocities determined by transmission measurements at cement cores. However, particularly for large (>2 m) tested structures such as GV2 here, the impact of the used velocity is significant as the depth information obtained from the imaging is highly dependent on the used velocity. Determining the shear wave velocities from the recorded ultrasonic data might sometimes provide other values in comparison to measurements at cores. Thus, we also performed tests with velocities derived from different methods and used additional information wherever possible, such as the dimensions of the analysed structures to verify the optimal velocity. Although reflectors can be imaged with a range of reasonable velocities, choosing an appropriate ultrasonic velocity is crucial to obtain reliable depth information from the obtained structural images.

### 4.3. Additional Data Analysis with Phase Analysis

With the research presented here, we also show the advantage of performing advanced data analysis. The phase analysis undertaken highlights the possibilities to not only identify reflectors in ultrasonic reflection data, but also analyse the potential cause of the reflector.

During the verification with drilling results, a unique correlation of every individual reflector to small-scale (cm–range) individual defects, such as cracks, is not possible. Due to the complexity of the underlying 3D reflections and inhomogeneous ultrasonic velocities occurring in the shotcrete, an unambiguous identification is challenging under realistic conditions. Nevertheless, larger structures, such as the significant delamination in core B33, are reasonably well associated with individual ultrasonic reflectors. Further on-going detailed comparison of cores and ultrasonic images of the whole analysed volume may provide more insights.

Thus, during the envisaged quality assurance, phase analysis may provide a method to decide if the identified significant structure is a threat (e.g., a delamination). Although extra time and know-how is needed, this provides extra knowledge and can be implemented in future analysis routines.

### 4.4. Application of Sophisticated Analysis Methods

To date, small-scale defects (3–8 cm size) are not reliably imaged, as shown in the experiment with artificial defects. Further investigations of advanced imaging techniques, allowing a better focus, are currently carried out. In combination with advanced measurement techniques, e.g., not relying on linear array instruments but on proper 3D transducer locations, the relative location of sender/receiver pairs might be more sensitive to specific defects within the concrete. Complementary data acquisition with a full 3D array was carried out at GSBV4 and still needs to be analysed in detail.

Advanced imaging techniques have already been successfully applied when imaging the Earth with seismic waves [25,26,27] but still need to be transferred to ultrasonic investigations in the field of non-destructive testing. For the reverse-time migration, this transfer has already successfully been accomplished for mass concrete [23,28,29]. Hence, methodological developments will further help to provide a reasonable quality assurance method using ultrasonic testing at engineered barrier systems.

Complementary investigations also aim to improve ultrasonic quality assurance at engineered barriers made from different salt concretes as mass concrete [30,31] but also show the capacity for great depth penetration of the LAUS ultrasonic system [16].

## 5. Conclusions

Engineered barrier systems made from magnesia concrete with salt aggregate and hard stone aggregate in a shotcrete procedure were examined concerning their suitability to enable safe nuclear waste disposal in an underground repository. Test sealing structures and smaller mock-ups have been built in the Teutschenthal mine and were analysed in detail. Particularly, a good connection between individual concrete sections is of interest.

In our study, despite the inhomogeneous internal reflectivity pattern of the shotcrete, the backwall (or opposite wall) of the small and large shotcrete constructions, as well as internal structures, such as boreholes, were detected and imaged using the ultrasonic echo method. Two different acquisition systems were applied and provide either great depth penetration (up to 9.6 m) with limited spatial resolution or detailed images of the upper ∼1 m. Although significant defects, such as an unexpected delamination, were clearly identified, smaller scale (cm–range) artificial defects were not reliably detected.

Advanced analysis methods, including phase analysis, can provide insight into the potential origin of the reflector. Significant reflectors were identified to correlate with weak zones and cracking, also seen in cores extracted from the blocks later. However, further improvements and combining more sophisticated measurement techniques with advanced imaging methods is expected to further improve the reliability and significance of the ultrasonic analysis.

Overall, ultrasonic methods provide means for quality assurance to identify internal structures and defects in magnesia shotcrete structures. To have a widely applicable methodology to ensure safe engineered barrier systems, it is crucial to further develop existing measurement and data analysis routines. Finally, ultrasonic non-destructive evaluation will thus contribute to develop and test safe technical barriers that are needed for safe nuclear waste disposal. The developed techniques will also be transferable to other waste types and applications.

## Figures and Tables

**Table 1:** Overview of the presented ultrasonic investigations at shotcrete experimental sites.

Experiment Name	Type	Construction Material	Shotcrete Layer Thickness	Ultrasonic Acquisition System
GV2	large-scale construction (z ≃ 10 m)	magnesia shotcrete with quartz aggregate	2.0…19.0 cm, mean of 9.9 cm	LAUS
GSBV3	shotcrete box (z ≃ 1 m)	magnesia shotcrete with salt aggregate	16.3…28.9 cm	linear array system
GSBV4	shotcrete box (z ≃ 1 m)	magnesia shotcrete with salt aggregate	∼16 cm	linear array system

## Data Availability

The ultrasonic data presented in this study are available on request from the corresponding author on reasonable cause.
