# Correction: Lay et al. Ultrasonic Quality Assurance at Magnesia Shotcrete Sealing Structures. *Sensors* 2022, *22*, 8717

**DOI:** 10.3390/s23187966

**Published:** 2023-09-19

**Authors:** Vera Lay, Ute Effner, Ernst Niederleithinger, Jennifer Arendt, Martin Hofmann, Wolfram Kudla

**Affiliations:** 1 Bundesanstalt für Materialforschung und Prüfung, 12205 Berlin, Germany; vera.lay@bam.de (V.L.); ute.effner@bam.de (U.E.); 2 Technical University Bergakademie Freiberg, 09596 Freiberg, Germany

The authors wish to correct the following errors in the original paper [1].

## Errors and Added Discussion in Text

During the analysis, we tested different constant velocities for the reconstruction of the ultrasonic echo data. Unfortunately, in the section “ultrasonic imaging at great depth” using two separate ultrasonic data sets, we mixed two different velocities in the presented results in the original paper [1] and wish to correct this error. We now plot all results from GV2 with the same velocity of v${}_{s}$(GV2) = 2430 m/s as previously written. All changes are marked in blue for the following specifications. Additionally, we also add a paragraph about ultrasonic velocities in the discussion.

- For the reflectors identified for the data from GV2, area 2, the description is changed as follows: “The deepest reflector is identified at a depth of z = −3.5 m (light blue arrow, 3) with a slightly shallower reflector located at z = −3.0 m (dark blue arrow, 2). The reflectors 2 and 3 can be attributed to the bearing of the barrier system in the rock mass or internal flaws and the opposite wall of the structure.”
- For the comparison of both ultrasonic measurements at GV2, the description is changed to the following: “In Figure 5a, the reflector 1 is associated with the bearing at y’ = −3.3 m, whereas reflector 3 might potentially be caused by 3D effects of the bearing or an internal flaw. Clearly, reflector 2 can be associated with the location (y’ = −9.6 m) of a pressure chamber at the end of the sealing structure. [...] Despite a slight local misalignment, reflector 5 might coincide with reflector 3, potentially caused by an internal flaw. Alternatively, reflector 5 might be associated with a borehole that was drilled after the initial ultrasonic measurements in area 1 (blue).”
- A discussion paragraph explaining the influence of ultrasonic velocities was added: “Generally, a constant ultrasonic shear wave velocity is used for the reconstruction of ultrasonic echo data assuming that the concrete’s elastic features are largely homogeneous. This approach usually provides robust results and is also used here with velocities determined by transmission measurements at cement cores. However, particularly for large (>2 m) tested structures such as GV2 here, the impact of the used velocity is significant as the depth information obtained from the imaging is highly dependent on the used velocity. Determining the shear wave velocities from the recorded ultrasonic data might sometimes provide other values in comparison to measurements at cores. Thus, we also performed tests with velocities derived from different methods and used additional information wherever possible, such as the dimensions of the analysed structures to verify the optimal velocity. Although reflectors can be imaged with a range of reasonable velocities, choosing an appropriate ultrasonic velocity is crucial to obtain reliable depth information from the obtained structural images.”

## Errors in Figures

- Figure 3a in [1]: Number denoting the length of the GV2 shotcrete sealing structure corrected to 10.25 m.
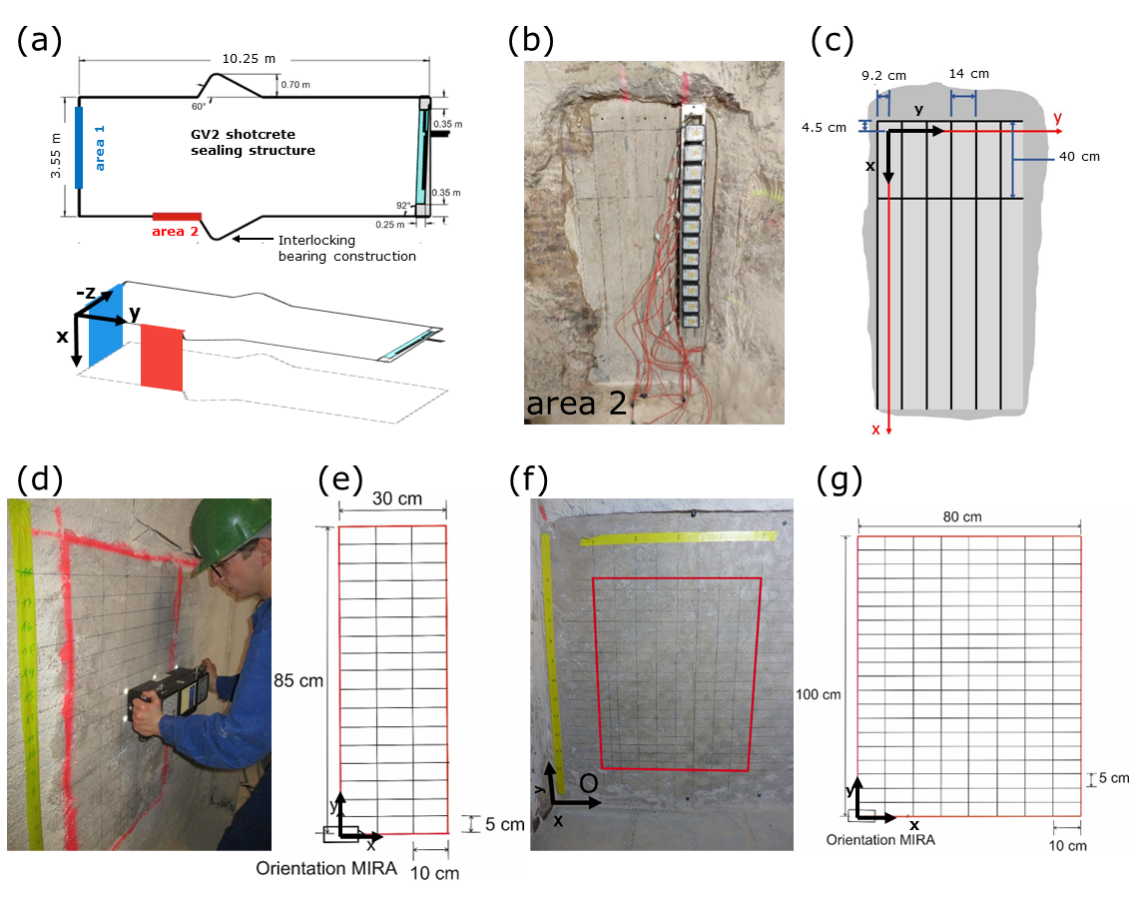
**Figure 3**. Ultrasonic data acquisition with (**a**–**c**) LAUS device and (**d**–**g**) array device. (**a**) Geometry GV2 experiment; (**b**) measurements at area 2 with LAUS; (**c**) corresponding measurement grid; (**d**) GSBV3 experiment with (**e**) corresponding measurement grid; (**f**) GSBV4 experiment with (**g**) corresponding measurement grid.
- Figure 4 in [1]: New plot with consistent analysis velocity of 2430 m/s. The caption is also corrected to refer to the relevant depth slice.
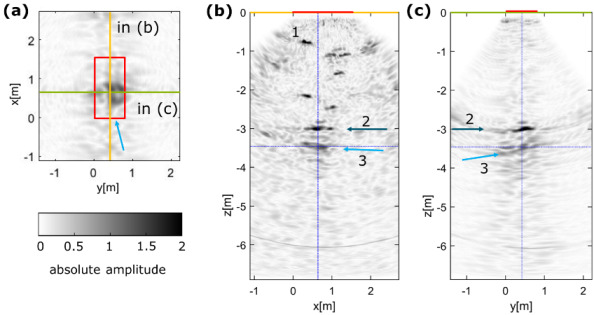
**Figure 4**. LAUS ultrasonic imaging results at area 2 at large-scale shotcrete construction. (**a**) xy–slice at a depth of z = −3.5 m with measurement area (red) and corresponding slices marked. [...]
- Figure 5 in [1]: We added the correct plot of Figure 5b using the same velocity. Additionally, we added the absolute amplitudes of both data sets as a legend and re-plotted Figure 5a in grey scale to have a more consistent figure. To highlight the shape of the engineered barrier, we changed the colour to orange.
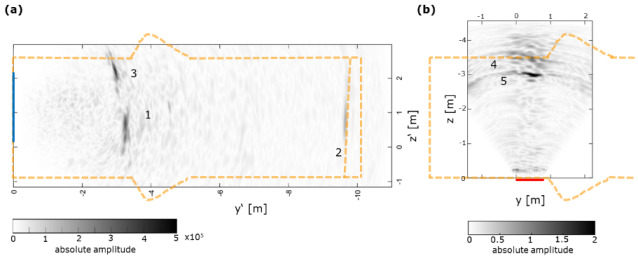
**Figure 5**. LAUS GV2 ultrasonic images and comparison to an engineered barrier (orange dashed line). [...]

The authors state that the scientific conclusions of the whole work are unaffected. This correction was approved by the Academic Editor. The original publication has also been updated.
